# Comparative Efficacy of Triple Versus Quadruple Therapy for the Eradication of *Helicobacter pylori* Infection in Asian Adults—A Systematic Review and Meta‐Analysis

**DOI:** 10.1002/jgh3.70292

**Published:** 2025-09-30

**Authors:** Abdur Razzak, Nymur Rahman, Nikkon Sarker, Monira Swapna Nil, Md. Arifur Rahman, Md. Toslim Mahmud

**Affiliations:** ^1^ Department of Microbiology Noakhali Science and Technology University Noakhali Bangladesh

**Keywords:** gastric disease, *Helicobacter pylori*, RCTs, systematic review and meta‐analysis

## Abstract

*Helicobacter pylori* infection remains a major health issue in Asia due to its strong association with gastric ulcers and cancer. Rising antibiotic resistance has reduced the success of standard triple therapy, leading to broader use of bismuth‐based quadruple regimens. However, the relative effectiveness of these regimens in Asian populations remains uncertain. To evaluate which treatment is more effective in Asian populations, this systematic review and meta‐analysis assessed randomized controlled trials comparing these two regimens. A comprehensive search across four major databases (PubMed, Web of Science, The Cochrane Library, and Scopus) up to May 2024 retrieved 16,398 studies. After thorough title and abstract (TIAB) and full‐text screening, nine eligible studies involving 2266 adult patients from six Asian countries were included for this analysis. The analysis showed that quadruple therapy achieved higher eradication rates than triple therapy within all nine studies, with a pooled risk ratio of 1.21 (95% CI: 1.09–1.35; *p* = 0.0003), indicating a 21% greater likelihood of treatment success. Although adverse effects were somewhat more frequent with quadruple therapy, adherence remained high in most trials. The overall risk of bias was low to moderate. These findings support the use of quadruple therapy as a more effective first‐line option for *H. pylori* eradication in Asia, especially in regions where clarithromycin resistance is common. Further research should focus on optimizing regimen tolerability and incorporating local antibiotic resistance patterns to guide treatment.

## 1 Introduction

*Helicobacter pylori* infection poses a substantial public health challenge, particularly in Asia, where it significantly contributes to the burden of gastric diseases, including gastritis, peptic ulcer disease, and gastric cancer [1, 2, 3, 4]. Generally, half of the world's population is infected with *pylori*, where the infection rate varies from 60% to 80% in developing countries and from 10% to 20% in developed countries [5]. Despite the progress in therapeutic approaches, the most effective strategy for *H. pylori* eradication remains a subject of debate, especially with the growing concern over increasing antibiotic resistance [6]. The increasing prevalence of multi‐drug resistant *H. pylori* and the genetic diversity of the pathogen have made the treatment of this infection more complex and subjective globally, which has led to an emphasis on the continual evaluation of existing treatment regimens and finding new and effective treatment options.

Traditionally, the first‐line treatment for *H. pylori* has been the triple therapy regimen, which comprises a proton pump inhibitor (PPI), clarithromycin, and amoxicillin, typically administered over 7 to 14 days [7, 8]. However, rising resistance to not only clarithromycin (exceeding 15%–20%), but also to metronidazole and levofloxacin has led to a decline in its efficacy, prompting the development and wider use of quadruple therapy regimens [9, 10]. These regimens, which add a bismuth salt, tetracycline, and either metronidazole or tinidazole to a PPI, aim to enhance eradication rates by expanding the antibiotic spectrum, incorporating bismuth for its synergistic effect and reduced dependence on a single antibiotic mechanism [11, 12]. Despite these advantages, bismuth‐containing quadruple therapy is not without limitations, including increased pill burden, gastrointestinal side effects, and limited accessibility to bismuth salts in some regions [13].

While global studies such as those by Luther et al. (2010) and Gene et al. (2003) have compared the efficacy of these regimens [14, 15], their findings may not be generalizable to Asian populations due to significant variations in *H. pylori* strain virulence factors, resistance profiles, host pharmacogenetics, dietary habits, and treatment adherence. Additionally, prior meta‐analyses have often pooled data from heterogeneous populations and lacked up‐to‐date regional resistance trends, limiting the reliability and applicability of their conclusions for the Asian context. Therefore, there is a critical need for a region‐specific synthesis of clinical evidence. This systematic review and meta‐analysis aim to critically evaluate and compare the efficacy of triple versus quadruple therapy for *H. pylori* infection in Asian adults. By synthesizing evidence from published randomized clinical trials, we will assess eradication rates, patient compliance, and adverse effects across different Asian populations, addressing critical gaps in knowledge and helping to identify the most effective treatment strategy for this region.

## 2 Methods

### 2.1 Literature Search Strategy

We have conducted this systematic review in accordance with the standard guidelines and reported the findings as per the Preferred Reporting Items for Systematic Reviews and Meta‐Analyses (PRISMA) [16]. The protocol was registered with PROSPERO (CRD42024550311).

A PICO table was constructed comprising population, intervention/exposure, comparator, and outcome (Data S1). A comprehensive literature search of four electronic databases including Medline through PubMed, Web of Science (core collection), The Cochrane Library (Cochrane Central Register of Controlled Trials), and Scopus was conducted to systematically identify relevant studies for this review. The search was focused on randomized controlled trials (RCTs) comparing the efficacy of triple versus quadruple therapy for *Helicobacter pylori* infection in adults from Asia. No restrictions on the date of publication were applied.

Search terms were developed using both Medical Subject Headings (MeSH) and free‐text keywords related to *Helicobacter pylori* infection, therapeutic regimens, and the target population. To ensure the retrieval of all relevant studies, Boolean operators (AND, OR) were employed to combine search terms. The terms used included: *“Helicobacter pylori”* OR “Helicobacter pylori infection” OR “Asian population” OR “Asia” OR “East Asian” OR “Southeast Asian” OR “South Asian” OR “Hp infection,” AND “Triple therapy” OR “Empirical” OR “Standard” OR “Triple therapy regimen” OR “Standard triple therapy” OR “PPI” OR “Amoxicillin” OR “Clarithromycin” OR “PPI‐based triple therapy” OR “Proton pump inhibitor,” AND “Quadruple therapy” OR “Quadruple treatment regimen” OR “Bismuth” OR “Tetracycline” OR “Metronidazole” OR “Bismuth quadruple therapy” OR “Bismuth salt” OR “Bismuth‐based quadruple treatment regimen,” AND “Eradication” OR “Eradication rate” OR “Treatment” OR “ *Helicobacter pylori* eradication” OR “Cure rate” OR “Adverse effects” OR “Side effects” OR “Negative conversion.” The keywords were adapted for different bibliographic databases by applying database‐specific filters. The retrieved records from the databases were downloaded and organized using the EndNote X8 software.

The search was completed on May 24, 2024. All retrieved articles were imported into Rayyan [17], a web‐based tool to streamline the screening and review process. Duplicate entries were removed based on the Rayyan report and careful review. The screening process was carried out in two phases. Initially, the titles and abstracts of the articles were screened, followed by a full‐text screening of selected articles. Two review authors worked separately on both phases. Two reviewers independently screened the titles and abstracts (TIAB) to determine eligibility, with conflicts similarly resolved through consensus. Afterward, full‐text articles were screened similarly.

### 2.2 Inclusion and Sequential Exclusion Criteria

The inclusion criteria for this review were: (1) studies involving *H. pylori* ‐infected adults aged 18 and above; (2) studies comparing the effectiveness of standard triple therapy (proton pump inhibitor [PPI], amoxicillin, and clarithromycin) versus standard quadruple therapy (PPI, bismuth salt, tetracycline, and metronidazole) in the eradication of *H. pylori* infection. Acceptable PPIs included omeprazole, esomeprazole, lansoprazole, rabeprazole, and pantoprazole; (3) Only articles published in English were considered for inclusion.

During the full‐text screening, a technique called “prioritization and sequential exclusion” was employed to determine the reasons for excluding articles [18]. In prioritizing studies for inclusion, we sequentially excluded those that (1) did not compare the standard triple therapy regimen (PPI, amoxicillin, clarithromycin) with the standard quadruple therapy regimen (PPI, bismuth salt, tetracycline, metronidazole); (2) studies that did not focus on *H. pylori* infection or related diseases; (3) those conducted outside of Asia; or (4) those categorized as case reports, case series, qualitative research, reviews, letters, opinions, conference papers, editorials, animal studies, or in vitro research were excluded. (5) Finally, non‐English studies were also omitted from consideration.

### 2.3 Data Extraction

Data extraction was performed independently by two reviewers using standardized forms created by the research team. These forms captured key study characteristics and outcomes, including the following variables: author name and publication year, sample size, study design, country, participant demographics (age, gender), treated disease, specific triple and quadruple therapy regimens, line of treatment, and treatment duration in days.

The primary outcome extracted was the eradication rate for both triple and quadruple therapy groups, while secondary outcomes included adverse events and patient compliance. The forms detailed the number of patients, number of cures, and cure rates (%) for both treatment groups under intention‐to‐treat (ITT) and per‐protocol (PP) analyses. Common side effects associated with each therapy were also documented. Where reported, relative risks (RR) and odds ratios (OR) with 95% confidence intervals, and *p*‐values for eradication rates were also extracted, along with compliance rates for the therapies. Any discrepancies between reviewers were addressed and resolved through discussion to reach consensus.

### 2.4 Assessment of the Risk of Bias in Included Studies

The risk of bias in the included studies was assessed using the Cochrane Risk of Bias tool for randomized controlled trials (RCTs) [19]. This tool evaluates several domains of bias, including selection bias, performance bias, detection bias, attrition bias, reporting bias, and other sources of bias [20].

Two reviewers independently conducted the assessment, where each study was assigned an overall risk of bias classification—low, moderate, or high—based on the assessments from the individual domains. Any disagreements between the reviewers were addressed through discussion, and, if necessary, a third reviewer was consulted to reach a consensus.

### 2.5 Statistical Analysis

The meta‐analysis was performed using Review Manager version 5.4 (Nordic Cochrane Centre, Cochrane Collaboration, Copenhagen, Denmark). For dichotomous outcomes, the Mantel–Haenszel method was used, and the pooled estimates of risk ratio (RR) with a 95% confidence interval (CI) were calculated for each study using a random‐effects model. A *p*‐value of less than 0.05 was considered statistically significant. The heterogeneity of the included studies was estimated using the standard chi‐square test and *I* ^2^ statistics.

## 3 Result

### 3.1 Articles Inclusion

**FIGURE 1:**
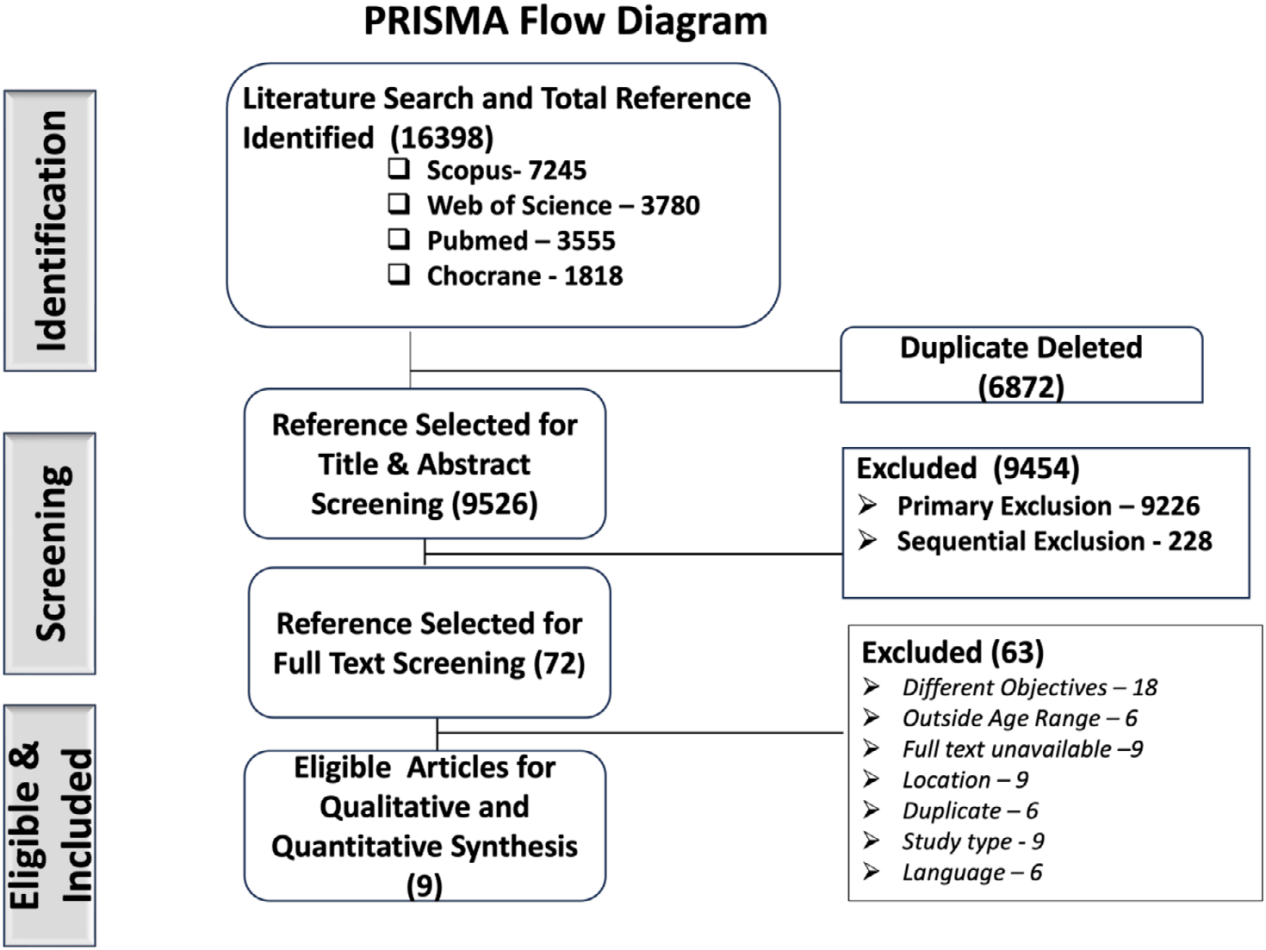
PRISMA flow diagram of the study selection process.

An initial keyword‐based search across four repositories identified 16,398 studies. These references were imported into the Rayyan platform for further processing, which involved duplicate removal and screening of titles and abstracts. Following this screening stage, 72 articles were selected for manual full‐text review. Of these, nine met all the inclusion criteria and were included in the study, while 63 did not meet the criteria and were excluded (Figure 1). The primary reasons for exclusion during the full‐text review were objectives other than stated in our protocol (18), followed by full‐text unavailability (9), location (09), articles type (09), age range (6), duplicate (6) and language (6). However, authors were requested for few of full‐text articles but not responded.

### 3.2 Baseline Characteristics of the Included Studies

All studies, published between 2010 and 2023, were conducted across six countries: South Korea (*n* = 2) [21, 22] Turkey (*n* = 3) [23, 24, 25], China (*n* = 1) [26], India (*n* = 1) [27], Iraq (*n* = 1) [28], and Taiwan (*n* = 1) [29]. All nine studies were randomized controlled trials (RCTs), either mentioned by authors or as per their study design, with eight described as single center and only one as multicenter [29]. The sample sizes varied considerably, ranging from 50 participants to 1080 participants [27, 28, 29] (Table 1).

**TABLE 1:** Baseline characteristics of the included articles.

Serial	Author (Year)	Therapy	Country	Study design	Sample size	Gender	Age	Disease treated	Diagnosis method	Treatment Regimen	Duration	Compliance
1.	G Gungor et al. [23]	Triple	Turkey	Single Center, Prospective, RCT	100	Male (39) Female (61)	≥ 18	*H. pylori* ‐related infection	Urea Breath, Stool Antigen, Rapid Urease, Histopathology Test	Pantoprazole 40 mg BID Amoxicillin 1 g BID Clarithromycin 500 mg BID	14	99%
		Quadruple			100	Male (43) Female (57)				Pantoprazole 40 mg BID, Bismuth subsalicylate 300 mg QID Tetracycline 500 mg QID Metronidazole 500 mg TID		96%
2	M Sezikli et al. [24]	Triple	Turkey	Single Center, Prospective, RCT	35	Male (16) Female (19)	18–79	*H. pylori* ‐related non‐ulcer dyspepsia	Histopathology, Rapid Urease, Urea Breath Test	Rabeprazole 20 mg BID Amoxicillin 1000 mg BID Clarithromycin 500 mg BID	14	NR
		Quadruple			35	Male (17) Female (18)				Rabeprazole 20 mg BID Bismuth QID Tetracycline 500 mg QID Metronidazole 500 mg TID		NR
3.	Q ZHENG et al. [26]	Triple	China	Single Center, Open, RCT	85	Male (30) Female (55)	18–70	*H. pylori* ‐related non‐ulcer dyspepsia	Urea Breath Test, Histopathology Test	Pantoprazole 40 mg BID Amoxicillin 1.0 g BID Clarithromycin 500 mg BID	7	NR
		Quadruple			85	Male (46) Female (39)				Pantoprazole 40 mg BID Bismuth 220 mg BID Tetracycline 750 mg BID Metronidazole 400 mg TID	10	NR
4.	JL Kim et al. [22]	Triple	South Korea	Single Center, Open, RCT	36	Male (24) Female (12)	20–80	Gastric MALT Lymphoma	Histopathology, Rapid Urease, Urea Breath Test	Esomeprazole 40 mg BID Amoxicillin 1 g BID Clarithromycin 500 mg BID	10	NR
		Quadruple			36	Male (17) Female (19)				Esomeprazole 40 mg BID Bismuth 300 mg BID Tetracycline 500 mg QID Metronidazole 500 mg TID		NR
5.	Panigrahi M.K et al. [27]	Triple	India	Single Center, Open, RCT	86	Male (58) Female (28)	18–80	*H. pylori* ‐related Gastrointestinal Disorders	Histological, Stool Antigen Test	Esomeprazole tablet 40 mg BID, Amoxicillin 1g BID, Clarithromycin tablet 500 mg BID	14	NR
		Quadruple			84	Male (65) Female (19)				Esomeprazole 40 mg BID, Tinidazole 500 mg BID, Bismuth 120 mg QID, Tetracycline 500 mg QID		NR
6.	Kim et al. [21]	Triple	South Korea	Single Center, Open, RCT	177	Male (83) Female (94)	≥ 18	*H. pylori* ‐related Gastrointestinal Disorders and Cancerous and Pre‐cancerous conditions	Urea Breath, Histological, Rapid Urease Test	Lansoprazole 30 mg BID Amoxicillin 1000 mg BID Clarithromycin 500 mg BID	7	88.4%
		Quadruple			175	Male (83) Female (92)				Lansoprazole 30 mg BID Tri potassium bismuth dicitrate 300 mg QID Tetracycline 500 mg, QID Metronidazole 500 mg TID	10	73.1%
7.	Liou JM et al. [29]	Triple	Taiwan	Multicenter, Open‐label, RCT	540	Male (267) Female (273)	≥ 20	Gastric Cancer and *H. pylori* infection	Urea Breath, Rapid Urease, Histology, Serology Test	Lansoprazole 30 mg, amoxicillin 1 g, clarithromycin 500 mg	14	85%
		Quadruple			540	Male (279) Female (261)				Lansoprazole 30 mg, bismuth tri potassium dicitrate 300 mg, tetracycline 500 mg, metronidazole 500 mg.	10	94%
8.	F Harmandar et al. [25]	Triple	Turkey	Single Center, RCT	40	Male (19) Female (21)	Adult as per Author	*H. pylori* infection with epigastric pain	Histological, Urea Breath Test	Pantoprazole 40 mg BID Amoxicillin 1 g BID Clarithromycin 500 mg BID.	14	NR
		Quadruple			40	Male (18) Female (22)				Pantoprazole 40 mg BID Bismuth salt 300 mg QID Tetracycline 500 mg QID Metronidazole 500 mg BID		NR
9.	A M Kadhim et al. [28]	Triple	Iraq	Single Center, Open, RCT	25	Male (13) Female (12)	20–65	*H. pylori* ‐related Gastrointestinal Disorders	Stool Antigen	Esomeprazole 40 mg BID Amoxicillin 1000 mg BID Clarithromycin 500 mg BID	14	NR
		Quadruple			25	Male (11) Female (14)				Esomeprazole 40 mg BID Bismuth subcitrate potassium 140 mg QID Tetracycline 125 mg QID Metronidazole 125 mg QID	7	NR

The demographic characteristics of the study populations were largely similar, with participants generally aged 18 or more in eight of the nine study [21, 22, 23, 24, 26, 27, 28], one remaining study did not report the age range [25], but stated that only adult patients were included. A variety of diagnostic methods were employed to diagnose and confirm *H. pylori* infection, including histopathology, rapid urease test (RUT), urea breath test (UBT), stool antigen test, and serology. The majority of studies utilized a combination of these methods. Notably, one study employed all four of the commonly used diagnostic tests (histopathology, RUT, UBT, and serology) [29], while another study relied solely on the stool antigen test for diagnosis [28].

Regarding the treatment regimens evaluated, all studies compared a triple therapy regimen with a quadruple therapy regimen. The backbone of the triple therapy across all studies consisted of a proton pump inhibitor (PPI), amoxicillin, and clarithromycin, while quadruple therapy combined a PPI with bismuth‐based compounds, tetracycline, and either metronidazole or tinidazole. The included studies exhibited variations in the duration of both triple and quadruple therapy regimens. A subset of studies implemented consistent treatment durations for both the triple and quadruple arms. A 14‐day duration for both triple and quadruple therapies was employed in four of the nine studies [23, 24, 25, 27]. JL Kim et al. opted for an intermediate duration of 10 days for both treatment arms [22]. In contrast, several studies investigated differing durations between the triple and quadruple regimens. Two studies utilized a 7‐day duration for the triple therapy arm while extending the quadruple therapy to 10 days [21, 26]. Liou JM et al. employed a 14‐day triple therapy regimen alongside a 10‐day quadruple therapy regimen [29]. Conversely, A M Kadhim et al. administered triple therapy for 14 days and quadruple therapy for a shorter 7‐day period [28]. The PPIs used in both therapies included esomeprazole in three studies [22, 27], pantoprazole in three studies [23, 25, 26], rabeprazole in only one study [24], and lansoprazole in two studies [21, 29]. A notable variation in the quadruple therapy regimen was observed in the study by Panigrahi MK et al., who utilized tinidazole as an alternative to metronidazole in their quadruple therapy arm [27].

Compliance and adherence to the treatment regimens were reported in three of the nine studies involving 1632 participants [21, 23, 29], rate of adherence to treatment is more than 85% in three of these studies, except for quadruple therapy in a study conducted by Kim et al. [21]. The highest adherence rates were reported by G Gungor et al. who reported more than 96% adherence [23].

### 3.3 Eradication Rate of *H. Pylori* and Adverse Outcomes

All included articles reported a higher eradication rate in quadruple therapy than in triple therapy (Table 2). Among all the articles where quadruple therapy showed a higher eradication rate, six of these studies reported statistically significantly improved (*p* < 0.05) eradication rates with quadruple therapy compared with triple therapy (77.5% vs. 48.3%; *p* < 0.001) [1], (91.6% vs. 65.1%; *p* < 0.05) [26], (97% vs. 81%; *p* = 0.046) [22], (92.9% vs. 70.1%; *p* < 0.001) [21], (96% vs. 88%; *p* < 0.0001) [29], (84% vs. 52%; *p* = 0.023) [28]. The other three studies, though reporting higher eradication rates for quadruple therapy, had *p*‐values more than 0.05 indicating statistically insignificant [24, 25, 27]. Both intention‐to‐treat (ITT) and per‐protocol (PP) analyses showed a similar trend.

**TABLE 2:** Outcomes of the included articles.

Serial	Author, year	Therapy	Primary outcome				Secondary outcome	*p*‐value
			ITT		PP			
			Participant	Cured % (*n*)	Participant	Cured % (*n*)	Adverse events (%)	
1.	G Gungor et al. [23]	Triple	100	42% (42)	87	48.3% (42)	NR	< 0.001
		Quadruple	100	62% (62)	80	77.5% (62)	NR	
2.	M Sezikli et al. [24]	Triple	35	51.40% (18)	35	51.40% (18)	40%	> 0.05
		Quadruple	30	56.70% (17)	27	62.9% (17)	14.8%	
3.	Q Zheng et al. [26]	Triple	85	63.5% (54)	83	65.1% (54)	60%	< 0.05
		Quadruple	85	89.40% (76)	83	91.6% (76)	42.30%	
4.	JL Kim et al. [22]	Triple	36	75% (27)	33	81.8% (27)	3.1%	0.046
		Quadruple	36	88.9% (32)	33	97% (32)	20%	
5.	Panigrahi M.K et al. [27]	Triple	99	80.8% (80)	86	93% (80)	NR	0.57
		Quadruple	98	82.65% (81)	84	96.4% (81)	NR	
6.	Kim et al. [21]	Triple	177	57.1% (101)	134	70.1% (94)	57.3%	< 0.001
		Quadruple	175	74.3% (130)	113	92.9% (105)	67.5%	
7.	Liou JM et al. [29]	Triple	540	83.7% (452)	508	88% (446)	47%	< 0.0001
		Quadruple	540	90.4% (488)	480	96% (461)	67%	
8.	F Harmandar et al. [25]	Triple	40	70% (28)	40	70% (28)	NR	> 0.05
		Quadruple	40	82.5% (33)	40	82.5% (33)	NR	
9.	A M Kadhim et al. [28]	Triple	25	52% (13)	25	52% (13)	64%	0.023
		Quadruple	25	84% (21)	25	84% (21)	72%	

For adverse events, six studies [21, 22, 24, 26, 28, 29] involving 1794 patients were analyzed. Four studies [21, 22, 28, 29] of the included six articles reported a higher incidence of adverse events in quadruple therapy than in triple therapy (20% vs. 3.1%) [22], (67.5% vs. 57.3%) [21], (67% vs. 47%) [29], (78% vs. 64%) [28]. The other two studies [24, 26] reported lower adverse events in the quadruple therapy arm compared to triple therapy (14.8% vs. 40%) [24], and (42.30% vs. 60%) [26].

### 3.4 Pooled Efficacy Analysis of Triple Versus Quadruple Therapy

**FIGURE 2:**
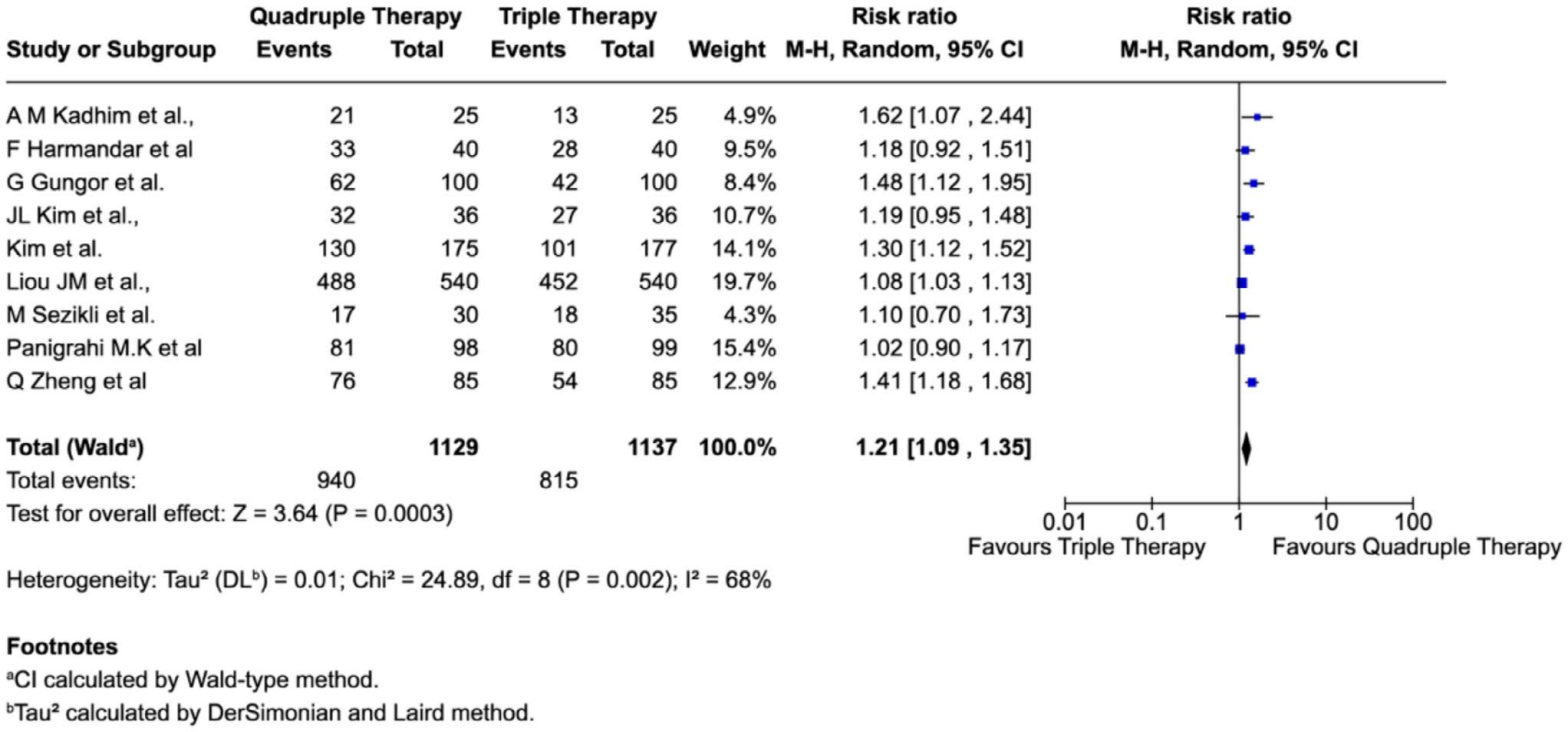
Forest plot of comparison of *H. pylori* eradication rate between quadruple therapy and triple therapy according to the ITT analysis.

The meta‐analysis of included nine studies showed a statistically significant efficacy of quadruple therapy over the standard triple therapy to eradicate the *H. pylori* infection among the Asian population. The pooled analysis for ITT, using a random‐effects model (Mantel–Haenszel method), yielded a risk ratio (RR) of 1.21 (95% CI: 1.09–1.35; *p* = 0.0003), indicating a 21% higher probability of eradication rate with quadruple therapy compared to triple therapy (Figure 2). The total sample included 2266 participants, where 940 events were cured among a total of 1129 events (83.26%) in the quadruple therapy group and 815 events were cured among a total of 1137 events (71.7%) in the triple therapy group. Significant moderate‐to‐high heterogeneity was observed across the studies (*I* ^2^ = 68%, *τ* ^2^ = 0.01, P for heterogeneity = 0.002), indicating some variability in treatment effects. Despite the observed heterogeneity, the overall direction of effect consistently favored quadruple therapy. These findings support the overall higher efficacy of quadruple therapy for *H. pylori* eradication. The similar trend was observed for the PP pooled analysis (data not shown).

### 3.5 Efficacy Across Treatment Durations and Regions (Subgroup Analysis)

**FIGURE 3:**
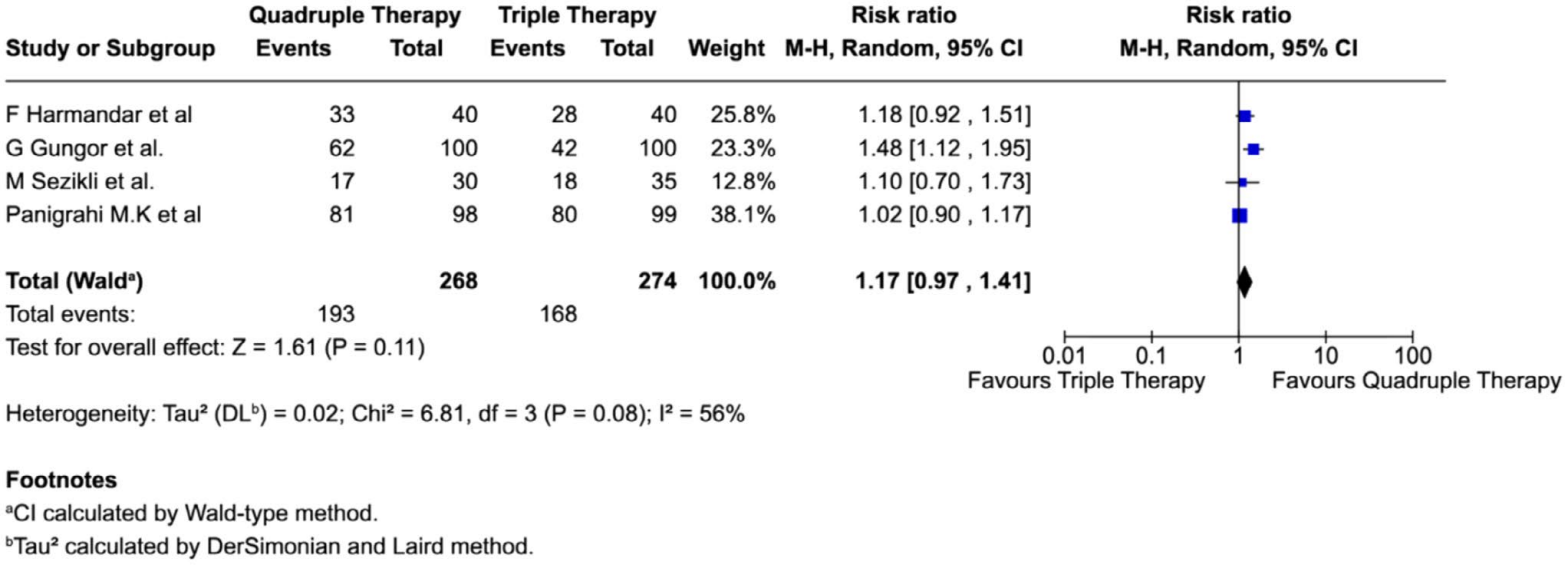
Subgroup analysis of *H. pylori* eradication comparing quadruple and triple therapies administered for 14 days.

Subgroup analyses were conducted to assess the impact of treatment duration and geographic region on eradication outcomes. When both triple and quadruple regimens were administered for 14 days, quadruple therapy showed a numerically higher eradication rate (~17% greater than triple therapy), although the difference did not reach statistical significance and moderate heterogeneity was noted (Figure 3).

**FIGURE 4:**
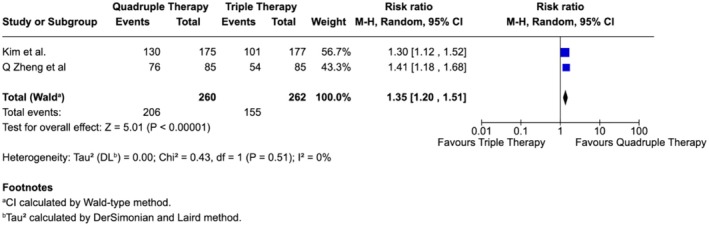
Subgroup analysis comparing *H. pylori* eradication between 7‐day triple therapy and 10‐day quadruple therapy.

In contrast, when shorter triple therapy (7 days) was compared with longer quadruple therapy (10 days), both studies demonstrated significantly superior eradication with quadruple therapy, and the pooled estimate confirmed this benefit without evidence of heterogeneity (0%) (Figure 4).

**FIGURE 5:**
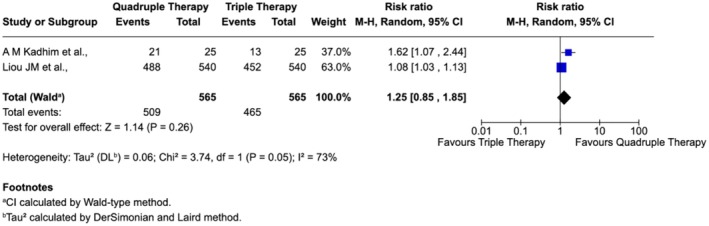
Subgroup analysis comparing *H. pylori* eradication between 14‐day triple therapy and 7–10‐day quadruple therapy.

Interestingly, even when triple therapy was extended to 14 days and compared against shorter quadruple regimens (7–10 days), quadruple therapy still yielded higher eradication rates, though the advantage was not statistically significant, suggesting regimen composition may be more critical than duration alone (Figure 5).

**FIGURE 6:**
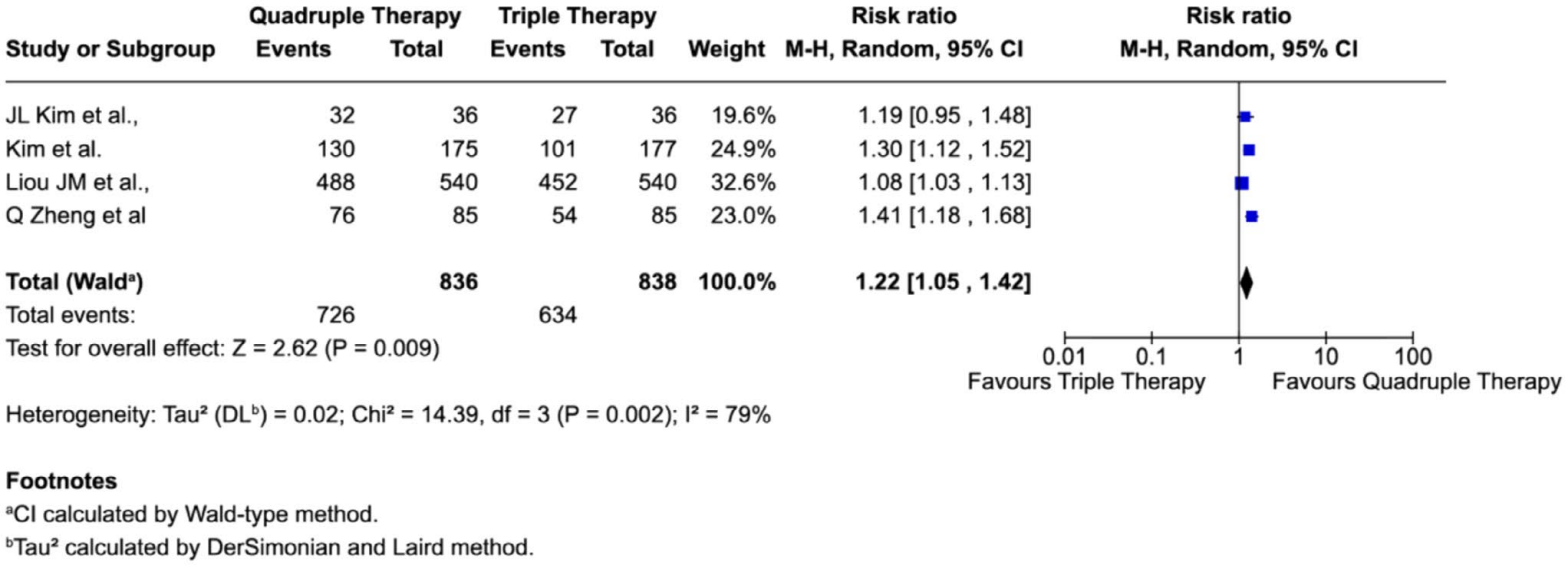
Subgroup analysis of *H. pylori* eradication comparing triple and quadruple therapy in East Asian countries.

Regional subgroup analysis further highlighted geographic variation in treatment efficacy. In East Asian countries (China, Korea, and Taiwan), quadruple therapy provided a significant benefit over triple therapy, with a pooled risk ratio of 1.22 (95% CI: 1.05–1.42), corresponding to a 22% higher eradication rate (Figure 6).

**FIGURE 7:**
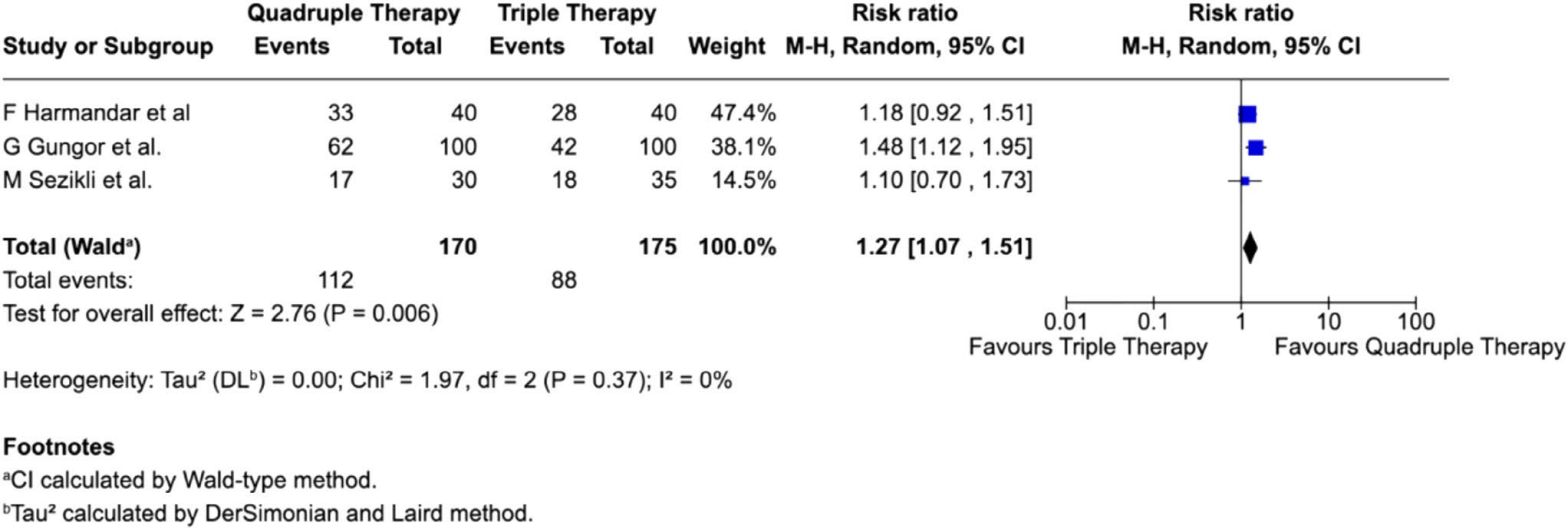
Subgroup analysis of *H. pylori* eradication comparing triple and quadruple therapy in Turkey.

However, this benefit was accompanied by considerable heterogeneity, indicating that local resistance patterns and clinical practices may influence outcomes. In contrast, evidence from West Asia (Turkey) was more consistent; across three studies, quadruple therapy demonstrated significantly higher eradication rates compared with triple therapy, with lower heterogeneity (Figure 7). Collectively, these findings suggest that quadruple therapy is generally more effective, though the magnitude of benefit may depend on regional resistance profiles and treatment duration.

### 3.6 Risk of Bias Assessment Across Included Studies

**FIGURE 8:**
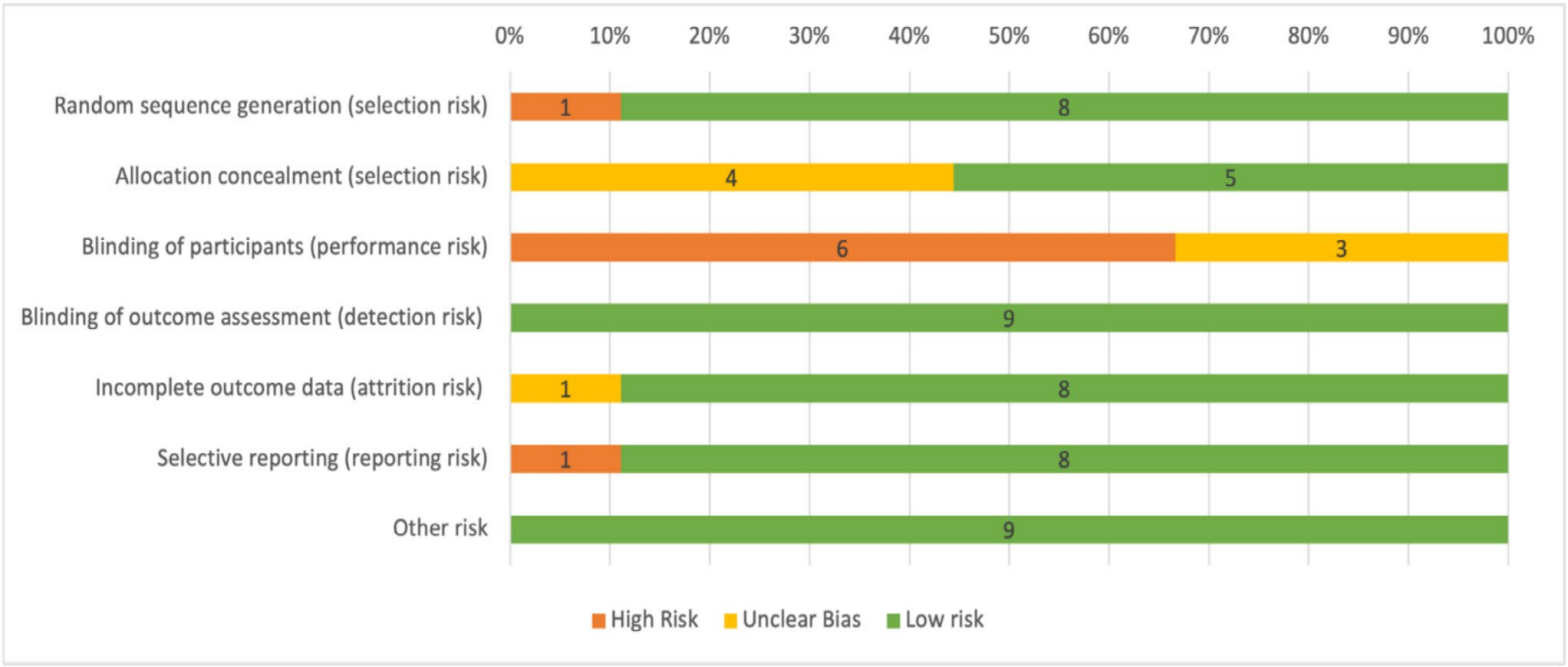
Risk of Bias assessment showing authors' judgment in percentage on each domain of risk of bias for all included studies.

Two independent reviewers conducted risk of bias assessments for the nine included studies. Overall, a low risk of bias was observed across most domains for the majority of studies. Random sequence generation was high risk in only one study [23] with the remaining eight at low risk [21, 22, 24, 25, 26, 27, 28, 29]. Allocation concealment was deemed low risk in five studies [21, 22, 24, 27, 29] and unclear in the remaining four studies [23, 25, 26, 28]. Due to open‐label study designs, performance bias was high in six studies [21, 22, 26, 27, 28, 29], low in one study [24], and unclear in two [23, 25]. Detection bias was consistently low across all included studies. Similarly, incomplete outcome data (attrition bias) presented a low risk in eight of the studies and an unclear risk in one [28]. Selective reporting was predominantly low risk (eight), with one study [27] assessed as high risk. Finally, all nine studies demonstrated a low risk of other biases. A visual summary of these assessments is provided in Figure 8.

## 4 Discussion

*Helicobacter pylori* infection remains a major public health concern in Asia, contributing to high rates of peptic ulcer disease and gastric cancer. The declining success of standard triple therapy, largely driven by increasing clarithromycin resistance, necessitates the need for alternative treatment strategies, based on region‐specific evidence. This systematic review and meta‐analysis critically evaluated randomized controlled trials (RCTs) comparing the efficacy of standard triple therapy versus quadruple therapy for *H. pylori* eradication in adult Asian populations.

Our findings contrast with earlier global meta‐analyses by Gené et al. (2003) and Luther et al. (2010), which reported no significant difference between triple and quadruple therapy. Several factors likely explain this divergence. First, both reviews pooled studies across diverse global populations, without accounting for local resistance patterns, strain variability, or treatment adherence factors that are particularly variable across Asia [30, 31]. Second, clarithromycin resistance has increased significantly in Asia over the past decade, reducing the efficacy of triple therapy and shifting clinical benefit in favor of bismuth‐containing quadruple regimens. Our analysis incorporates more recent RCTs that better reflect current resistance patterns and prescribing practices. The observed superiority of quadruple therapy in our review is consistent with evolving global treatment guidelines, which recommend it as the first‐line option in regions where clarithromycin resistance exceeds 15% [32, 33]. Across all nine included studies, eradication rates with quadruple therapy were uniformly higher than those achieved with triple therapy, with six trials demonstrating statistically significant superiority. Notably, the largest and most statistically influential study, conducted by Liou et al. 2016, enrolled 1080 participants and contributed 19.7% of the total meta‐analytic weight, reported a modest but statistically significant benefit of quadruple therapy (RR: 1.08, 95% CI: 1.02–1.14; *p* < 0.0001) with low risk of bias [29]. This result aligns directionally with the overall pooled estimate (RR: 1.21, 95% CI: 1.09–1.35; *p* = 0.0003), confirming the robust superiority of quadruple therapy. The magnitude of this difference was often clinically meaningful, with several studies reporting absolute increases in eradication exceeding 20%. For instance, in the study by Gungor et al. (2015), quadruple therapy achieved a per‐protocol (PP) eradication rate of 77.5% versus only 48.3% for triple therapy (*p* < 0.001), while Kim et al. (2021) reported rates of 92.9% versus 70.1% (*p* < 0.001), representing an absolute increase of 22.8%. These findings are in line with the established understanding that triple therapy, originally the global first‐line standard, has experienced a sharp decline in effectiveness due to rising clarithromycin resistance, particularly in Asia, where resistance rates now frequently exceed 20%–30% [34, 35, 36]. In the case of quadruple therapy, the addition of bismuth and the use of multiple antimicrobials, including tetracycline and metronidazole, likely mitigates the risk of treatment failure due to single‐drug resistance, offering a pharmacologically broader spectrum compared to clarithromycin‐based triple therapy [37].

However, the extent of quadruple therapy's benefit varied across studies and countries, reflecting underlying microbial ecology and antibiotic stewardship practices [38, 39]. The observed heterogeneity in treatment effect (*I* ^2^ = 68%) strongly suggests that therapeutic outcomes vary meaningfully across Asian subregions. Subgroup analyses confirmed this variability. In East Asia (China, Korea, Taiwan), quadruple therapy consistently demonstrated a significant advantage, with a pooled risk ratio of 1.22, corresponding to a 22% higher eradication rate than triple therapy. Yet, substantial heterogeneity persisted. In contrast, findings from West Asia (Turkey) were more consistent: across three studies, quadruple therapy provided a clear and statistically significant benefit with lower heterogeneity. By comparison, South Asian evidence was sparse, but the available data suggested only marginal differences between regimens, with one Indian trial reporting comparable efficacy [27]. These discrepancies may stem from local variations in *H. pylori* resistance to metronidazole and tetracycline [40], differences in bacterial genotypes [41], or host‐related factors such as CYP2C19 polymorphisms influencing PPI metabolism [42]. Additionally, inconsistencies in diagnostic confirmation, for instance, reliance solely on stool antigen test vs. urea breath test or histopathology might have influenced reported eradication outcomes and underscored the importance of uniform diagnostic criteria.

A key variable assessed across studies was treatment duration, which has long been recognized as an important determinant of eradication success. However, the subgroup analysis indicates that the superior efficacy of quadruple therapy is not solely a function of treatment length. Even when triple therapy was administered for longer periods, quadruple regimens maintained higher eradication rates, highlighting the importance of regimen composition and synergistic antimicrobial activity over duration alone. Comparisons between shorter triple regimens and longer quadruple courses consistently favored the latter, demonstrating that quadruple therapy can achieve robust eradication even with abbreviated schedules. These findings indicate that while extended treatment durations (10–14 days) are generally associated with improved outcomes, the composition and potency of the regimen may have a greater impact than duration alone.

A recurring concern with quadruple therapy is its complex regimen, often associated with a higher pill burden and risk of adverse events, potentially undermining adherence [43]. While our review found > 85% adherence in most studies that reported this outcome, the data were sparse; only three of nine studies provided compliance metrics. Notably, Kim et al. documented reduced adherence in the quadruple arm, underscoring the clinical trade‐off between efficacy and patient tolerance. The spectrum of reported adverse events, ranging from gastrointestinal disturbances to bitter taste and darkened stool, highlights the need for careful patient counseling and regimen personalization [44]. Simplified bismuth‐based formulations or the use of tetracycline alternatives such as doxycycline may improve tolerability and compliance in future interventions [45].

While all included studies were RCTs, they exhibited substantial heterogeneity in methodology, diagnostic approaches, treatment duration, and geographic settings. Risk of bias was generally low to moderate, yet the predominance of single‐center studies limits generalizability [46]. Moreover, the absence of antimicrobial susceptibility testing in most trials represents a critical omission; empirical regimens were used without stratification based on clarithromycin or metronidazole resistance [47]. Future studies incorporating pretreatment resistance profiling are essential for guiding individualized therapy and informing region‐specific guidelines.

This review is not without limitations. The relatively small number of RCTs directly comparing the two regimens in Asian populations restricts broader applicability. Most trials were conducted in East and West Asian countries (South Korea, China, Taiwan, and Turkey) with additional representation from South Asia (India) and Western Asia (Iraq). However, large regions such as Southeast Asia (including Indonesia, Thailand, Vietnam), Central Asia (including Kazakhstan, Uzbekistan), and other parts of South Asia remain underrepresented. Moreover, the exclusion of a few potentially relevant studies is due to full‐text inaccessibility. Despite retrieval attempts including institutional access and contacting corresponding authors, the full texts could not be obtained, which may introduce a minor risk of selection bias. Additionally, language bias may have occurred due to the exclusion of non‐English studies. Variations in outcome reporting (PP vs. ITT), diagnostic methods, and compliance metrics introduced heterogeneity that complicates cross‐study comparison.

From a clinical standpoint, while directionally consistent with the global picture, our study highlights the importance of country or region‐specific research to optimize antibiotic use and minimize resistance. Policymakers and national gastroenterology associations should prioritize local antimicrobial surveillance to tailor empiric regimens and improve eradication outcomes. Moreover, investment in fixed‐dose combination therapies and simplified dosing schedules may enhance adherence and mitigate the complexity of quadruple regimens.

Future RCTs should incorporate antibiotic susceptibility testing, pharmacogenomic profiling, and standardized outcome measures. Studies evaluating novel regimens, such as concomitant or sequential therapy, or emerging non‐antibiotic‐based approaches (probiotics, antimicrobial peptides, or vaccine candidates), also merit attention. Additionally, real‐world data from multicenter, longitudinal cohorts will be vital in understanding long‐term eradication success, reinfection rates, and post‐treatment complications such as dysbiosis or antibiotic resistance amplification.

In conclusion, we summarized available data and our systematic review and meta‐analysis demonstrated that the effective treatment of *H. pylori* infection among the Asian population is bismuth salt‐based quadruple therapy, which is not different from the global trend. However, there is substantial variation in the *H. pylori* prevalence rate and antibiotic resistance pattern in Asian countries compared with European and American countries. Therefore, future RCTs should incorporate antibiotic susceptibility testing, pharmacogenomic profiling, and standardized outcome measures. Studies evaluating novel regimens, such as concomitant or sequential therapy, or emerging non‐antibiotic‐based approaches (probiotics, antimicrobial peptides, or vaccine candidates), also merit attention. Additionally, real‐world data from multicenter, longitudinal cohorts will be vital in understanding long‐term eradication success, reinfection rates, and post‐treatment complications such as dysbiosis or antibiotic resistance amplification.

## Conflicts of Interest

The authors declare no conflicts of interest.

## Supporting information


**Data S1:** Supporting Information.

## Data Availability

The data that supports the findings of this study is available in the Supporting Information of this article.
