# Supplementary material for: Comparative Efficacy of Triple Versus Quadruple Therapy for the Eradication of Helicobacter pylori Infection in Asian Adults—A Systematic Review and Meta‐Analysis
Source: JGH Open. 2025 Sep 30;9(10):e70292. doi: 10.1002/jgh3.70292 (PMC12483952; doi:10.1002/jgh3.70292)
Supplement: Supplementary file 1 — Data S1: Supporting Information. [file JGH3-9-e70292-s001.docx]

**Search has done 24 May, 2024**

**1. Comprehensive search strategy: PubMed format**

Number of studies obtained: **3555.**

("Helicobacter pylori"[All Fields] OR "Helicobacter pylori Infection"[All Fields] OR "Asian population"[All Fields] OR "Asia"[All Fields] OR "East Asian"[All Fields] OR "Southeast Asian"[All Fields] OR "South Asian"[All Fields] OR "Hp infection"[All Fields]) AND ("Triple therapy"[All Fields] OR "empirical"[All Fields] OR "standard"[All Fields] OR "Triple therapy regimen"[All Fields] OR "Standard triple therapy"[All Fields] OR "PPI"[All Fields] OR "amoxicillin"[All Fields] OR "clarithromycin"[All Fields] OR "PPI-based triple therapy"[All Fields] OR "Proton pump inhibitor"[All Fields]) AND ("Quadruple therapy"[All Fields] OR ((("quadruple"[All Fields] OR "quadruples"[All Fields]) AND ("clinical protocols"[MeSH Terms] OR ("clinical"[All Fields] AND "protocols"[All Fields]) OR "clinical protocols"[All Fields] OR ("Treatment"[All Fields] AND "regimen"[All Fields]) OR "treatment regimen"[All Fields])) OR ("bismuth"[MeSH Terms] OR "bismuth"[All Fields] OR "bismuthate"[All Fields] OR "bismuthates"[All Fields])) OR "tetracycline"[All Fields] OR "metronidazole"[All Fields] OR "bismuth quadruple therapy"[All Fields] OR "Bismuth salt"[All Fields] OR ("bismuth based"[All Fields] AND ("quadruple"[All Fields] OR "quadruples"[All Fields]) AND ("clinical protocols"[MeSH Terms] OR ("clinical"[All Fields] AND "protocols"[All Fields]) OR "clinical protocols"[All Fields] OR ("Treatment"[All Fields] AND "regimen"[All Fields]) OR "treatment regimen"[All Fields]))) AND ("Eradication"[All Fields] OR "Eradication rate"[All Fields] OR "Treatment"[All Fields] OR "Helicobacter pylori eradication"[All Fields] OR "cure rate"[All Fields] OR "Adverse effects"[All Fields] OR "Side effects"[All Fields] OR "Negative conversion"[All Fields])

**2. Comprehensive search strategy: Scopus**

Number of studies obtained: **7245**

( TITLE-ABS-KEY ( "Helicobacter pylori"  OR  "Helicobacter pylori Infection"  OR  "Asian population"  OR  "Asia"  OR  "East Asian"  OR  "Southeast Asian"  OR  "South Asian"  OR  "Hp infection" )  AND  TITLE-ABS-KEY ( "Triple therapy"  OR  "empirical"  OR  "standard"  OR  "Triple therapy regimen"  OR  "Standard triple therapy"  OR  "PPI"  OR  "amoxicillin"  OR  "clarithromycin"  OR  "PPI-based triple therapy"  OR  "Proton pump inhibitor" )  AND  TITLE-ABS-KEY ( "Quadruple therapy"  OR  "Quadruple treatment regimen"  OR  "bismuth"  OR  "tetracycline"  OR  "metronidazole"  OR  "bismuth quadruple therapy"  OR  "Bismuth salt"  OR  "bismuth-based quadruple treatment regimen" )  AND  TITLE-ABS-KEY ( "Eradication"  OR  "Eradication rate"  OR  "Treatment"  OR  "Helicobacter pylori eradication"  OR  "cure rate"  OR  "Adverse effects"  OR  "Side effects"  OR  "Negative conversion" ) )

**3. Comprehensive search strategy: Web of Science**

Number of studies obtained: **3780**

(((ALL=("Helicobacter pylori" or "Helicobacter pylori Infection" OR "Asian population" OR "Asia" OR "East Asian" OR "Southeast Asian" OR "South Asian" OR “Hp infection” )) AND ALL=("Triple therapy" OR “empirical” OR “standard” OR "Triple therapy regimen" OR "Standard triple therapy" OR   "PPI” OR “amoxicillin” OR “clarithromycin" OR "PPI-based triple therapy" OR "Proton pump inhibitor")) AND ALL=("Quadruple therapy" OR "Quadruple treatment regimen" OR “bismuth” OR “tetracycline” OR “metronidazole" OR "bismuth quadruple therapy" OR "Bismuth salt" OR "bismuth-based quadruple treatment regimen")) AND ALL=("Eradication" OR "Eradication rate" OR "Treatment" OR "Helicobacter pylori eradication" OR "cure rate" OR "Adverse effects" OR "Side effects" OR "Negative conversion")

**4. Comprehensive search strategy Cochrane Library**

Number of studies obtained**: 1818**

"Helicobacter pylori" or "Helicobacter pylori Infection" OR "Asian population" OR "Asia" OR "East Asian" OR "Southeast Asian" OR "South Asian" OR “Hp infection” in Title Abstract Keyword AND "Triple therapy" OR “empirical” OR “standard” OR "Triple therapy regimen" OR "Standard triple therapy" OR "PPI” OR “amoxicillin” OR “clarithromycin" OR "PPI-based triple therapy" OR "Proton pump inhibitor" in Title Abstract Keyword AND "Quadruple therapy" OR "Quadruple treatment regimen’ OR bismuth” OR “tetracycline” OR “metronidazole" OR "bismuth quadruple therapy" OR "Bismuth salt" OR "bismuth-based quadruple treatment regimen" in Title Abstract Keyword AND "Eradication" OR "Eradication rate" OR "Treatment" OR "*Helicobacter pylori* eradication" OR "cure rate" OR "Adverse effects" OR "Side effects" OR "Negative conversion" in Title Abstract Keyword

**Excluded articles with reasons:**

| Sl No. | Title of the Articles | Reason for exclusion |
| --- | --- | --- |
| 1 | What is the impact of density on the success of eradication therapy: A clinico-histopathological study | Outside age range |
| 2 | Low eradication rate of with triple 7-14 days and quadruple therapy in Turkey | Outside age range |
| 3 | Comparative study of eradication rates of “concomitant therapy" versus "bismuth containing metronidazole-based triple therapy" | Language |
| 4 | Quadruple Therapy with Bismuth Subcitrate Potassium, Metronidazole, Tetracycline, and Omeprazole is Superior to Triple Therapy with Omeprazole, Amoxicillin, and Clarithromycin in the Eradication of *Helicobacter pylori* | Full-text unavailable |
| 5 | 10-Days Concomitant Therapy, 10-Day Bismuth Quadruple Therapy, and 14-Day Triple Therapy in the First Line Treatment of Infection - A Multicenter Randomized Trial | Full-text unavailable |
| 6 | Eradication of H pylori infection in a rural population: One-day quadruple therapy versus 7-day triple therapy | Different objectives |
| 7 | Proton pump inhibitor based triple therapy combined with bismuth is superior to proton pump inhibitor based triple therapy only for eradication.: (Preliminary data) | Full-text unavailable |
| 8 | Quadruple Therapy Is Superior to Triple Therapy in the Treatment of Clarithromycin Exposed | Article type: conference paper |
| 9 | The efficacy of bismuth containing quadruple therapy as a first-line treatment option for *Helicobacter pylori* | Study location not mentioned |
| 10 | One-week triple vs. quadruple therapy for *Helicobacter pylori* infection — a randomized trial | Study location not mentioned |
| 11 | A randomized comparison of quadruple and triple therapies for *Helicobacter pylori* eradication: The QUADRATE Study | Study location |
| 12 | Comparison of eradication rates between 7 and 14 days of tailored therapy according to clarithromycin resistance test: A randomized, multicenter, non-inferiority study | Different objectives |
| 13 | Empirical *Helicobacter pylori* “rescue” therapy after failure of two eradication treatments | Study location |
| 14 | Evaluation of the efficacy and safety of the hybrid scheme for eradication therapy of *Helicobacter pylori* infection | Language |
| 15 | *Helicobacter pylori* eradication as the sole treatment for gastric and duodenal ulcers | Study location |
| 16 | Eradication of *Helicobacter pylori* by quadruple therapy at ordinary doses once daily for one week - Comparison with triple therapy, reasons for efficacy of MINO combination and the secondary drug-resistance | Full-text unavailable |
| 17 | Empiric *H. pylori* therapy—10-day concomitant, bismuth quadruple or 14-day triple therapy: none is best | Study type: commentray/ review paper |
| 18 | Effectiveness of triple therapy and quadruple therapy for *Helicobacter pylori* eradication | Language |
| 19 | Treatment options for *Helicobacter pylori* infection when proton pump inhibitor-based triple therapy fails in clinical practice | Study location |
| 20 | Efficacies of Susceptibility-guided vs Empiric Therapy for Rescue Treatment of *Helicobacter Pylori* Infection | Study type: clinical trail |
| 22 | A Randomized Comparison of Quadruple and Triple Therapies for Helicobacter pylori Eradication: The QUADRATE Study | Duplicate |
| 23 | Comparison of 7-day PPI-based Standard Triple Therapy and 10-day Bismuth Quadruple Therapy for *H. Pylori* Eradication | Study type: clinical trail |
| 24 | Bismuth-Based First-Line Therapy for *Helicobacter pylori* Eradication in Type 2 Diabetes Mellitus Patients | Different objectives |
| 25 | A Randomized Trial of Lansoprazole, Amoxycillin, and Clarithromycin versus Lansoprazole, Bismuth, Metronidazole and Tetracycline in the Retreatment of Patients Failing Initial *Helicobacter pylori* Therapy | Study location |
| 26 | *Helicobacter pylori* eradication as the sole treatment for gastric and duodenal ulcers | Study location |
| 27 | The Efficacy of the 7 Days Tailored Therapy as the 1st Eradication of *H. Pylori* Infection | Study type: clinical trail |
| 28 | Comparison of the efficacy of pantoprazole-based triple therapy versus quadruple therapy in the treatment of *Helicobacter pylori* infection: a Single-center, randomized, open and parallel-controlled study | Different objectives |
| 29 | Comparison of *Helicobacter pylori* eradication rates between 7 and 14 days of tailored therapy according to clarithromycin resistance test: A randomized, multicenter, non-inferiority study | Duplicate |
| 30 | Triple Therapy Versus Quadruple Therapies in the First Line Therapy of *Helicobacter Pylori* Infection | Duplicate |
| 32 | 10-day concomitant therapy, 10-day bismuth quadruple therapy, and 14-day triple therapy in the first-line treatment of Helicobacter pylori infection-a multicenter randomized trial | Duplicate |
| 33 | Comparison of Vonoprazan-based Versus Lansoprazole-based Triple Therapy, High Dose Dual Therapy, Bismuth and Non-bismuth Quadruple Therapy in the First-line Treatment of *Helicobacter Pylori* Infection | Different objectives |
| 34 | Efficacy analysis of empirical bismuth quadruple therapy, high-dose dual therapy, and resistance gene-based triple therapy as a first-line *Helicobacter pylori* eradication regimen – An open-label, randomized trial | Different objectives |
| 35 | One-week triple vs. quadruple therapy for Helicobacter pylori infection — a randomized trial | Duplicate |
| 36 | Quadruple therapy versus standard triple therapy for eradication of *Helicobacter pylori* in Kuwait | Outside age range |
| 37 | Five Days Quadruple and Clarithromycin Containing Triple Therapy as Treatment for *Helicobacter Pylori* Eradication | Full-text unavailable |
| 39 | Comparing the efficacy of four different protocols for eradicating of *Helicobacter pylori* infection in Ahvaz, southwest Iran | Different objectives |
| 40 | COMPARISON of EFFECTIVENESS of STANDARD TRIPLE THERAPY VERSUS BISMUTH-BASED THERAPY for the MANAGEMENT of PEPTIC ULCER DISEASE DUE to HELICOBACTER PYLORI | Different objectives |
| 41 | Comparison of the efficacy of different treatment regimen for treating stomach infection by *Helicobacter pylori* | Full-text unavailable |
| 42 | Initial trials with susceptibility-based and empiric anti-*H. Pylori* Therapies in Mongolia | Different objectives |
| 44 | Comparison of Empirical and Genotypic Resistance-Guided Tailored Therapy for *Helicobacter pylori* Infection: a Randomized Controlled Trial | Different objectives |
| 45 | Effect of tailored *Helicobacter pylori* eradication with platelet count recovery in immune thrombocytopenic purpura | Different objectives |
| 46 | Erratum: helicobacter pylori eradication with a capsule containing bismuth subcitrate potassium, metronidazole, and tetracycline given with omeprazole versus clarithromycin-based triple therapy: a randomised, open-label, non-inferiority, phase 3 trial | Study location |
| 47 | 1 day quadruple therapy was not inferior to 7 day triple therapy for eradication of Helicobacter pylori infection in dyspepsia | Study type: Summary |
| 48 | Efficacy of Culture-Based Tailored Therapy as Primary Treatment of *Helicobacter pylori* Infection: a Multicenter Prospective Study | Study type: Conference |
| 49 | Quadruple therapy for initial eradication of *Helicobacter pylori* in peptic ulcer: comparison with triple therapy | Study type: review paper |
| 50 | Study for the efficacy and influencing factors of different protocols in the treatment of *Helicobacter pylori* eradication | Different objectives |
| 51 | *'Helicobacter pylori'* treatment: comparison of two therapeutic schemes | Full-text unavailable |
| 52 | Triple or quadruple tetracycline-based therapies versus standard triple treatment for *Helicobacter pylori* treatment | Outside age range |
| 53 | Omeprazole-based triple and Bismuth containing quadruple therapy for *Helicobacter pylori* eradication in patients with chronic gastritis: a prospective randomized study in Zhejiang, China | Different objectives |
| 55 | Comparison of the effectiveness of tri- and quadricomponent eradication therapy in patients with peptic ulcer | Language |
| 56 | A multi-center, randomized controlled study on the effect of Saccharomyces boulardii combined with triple therapy for the initial eradication of *Helicobacter pylori* infection | Language |
| 57 | Comparison of the efficacies of standard triple, sequential and quadruple antibiotic therapy in eradicating *Helicobacter pylori* infection: a randomized controlled trial | Duplicate |
| 58 | EFFICACY OF CULTURE-BASED TAILORED THERAPY AS PRIMARY TREATMENT OF *HELICOBACTER PYLORI* INFECTION: PRELIMINARY REPORT OF A MULTICENTER PROSPECTIVE STUDY | Full-text unavailable |
| 59 | Comparison of the efficacy of triple versus quadruple therapy on the Chinese patients with chronic gastritis | Full-text unavailable |
| 60 | First-line therapy in *Helicobacter pylori* eradication therapy: experience of a surgical clinic. | Language |
| 62 | One-day quadruple therapy compared with 7-day triple therapy for Helicobacter pylori infection | Different objectives |
| 64 | What is the impact of *Helicobacter pylori* density on the success of eradication therapy: a clinico-histopathological study. | Outside age range |
| 66 | High rate of post-therapeutic resistance after failure of macrolide-nitroimidazole triple therapy to cure *Helicobacter pylori* infection: impact of two second-line therapies in a randomized study. | Different objectives |
| 67 | Low eradication rate of Helicobacter pylori with triple 7-14 days and quadriple therapy in Turkey. | Outside age range |
| 68 | Ten-day empirical sequential or concomitant therapy is more effective than triple therapy for *Helicobacter pylori* eradication: A multicenter, prospective study. | Different objectives |
| 69 | 1-day quadruple therapy was not inferior to 7-day triple therapy for eradication of *Helicobacter pylori* infection in dyspepsia. | Study type: Summary |
| 71 | Triple drug therapy with proton pump inhibitor a better option for *helicobacter pylori* eradication | Different objectives |
| 72 | Eradication therapy in Helicobacter pylori-positive patients with halitosis: Long-term outcome | Different objectives |

**PICO table:**

| Population | **AND**  Intervention | **AND….**  Comparator | **AND**  Outcome |
| --- | --- | --- | --- |
| Asian countries; Age group >18, both male and female; *Helicobacter pylori* infected patients | Triple therapy (PPI, amoxicillin, and clarithromycin) | Quadruple therapy (proton pump inhibitor, bismuth salt, tetracycline, and metronidazole or tinidazole) | Eradication rate; adverse effects. |
